# Associations of Cervical Features and Preventive Interventions With Gestational Age at Delivery

**DOI:** 10.7759/cureus.111857

**Published:** 2026-06-30

**Authors:** Christina Pagkaki, Nektaria Kritsotaki, Anastasia Bothou, Vasiliki Kourti, Georgios Tsatsaris, Eftimios Oikonomou, Nikolaos Machairiotis, Nick Tsikouras, Rath Werner, Anastasia Grapsa, Nikoletta Koutlaki, Panagiotis Tsikouras

**Affiliations:** 1 Department of Obstetrics and Gynecology, University General Hospital of Alexandroupolis, Alexandroupolis, GRC; 2 Department of Midwifery, University of West Attica, Athens, GRC; 3 Third Department of Obstetrics and Gynecology, Attiko University Hospital, National and Kapodistrian University of Athens School of Medicine, Athens, GRC; 4 Department of Obstetrics and Gynecology, Universitätsklinikum Aachen, Aachen, DEU; 5 Department of Microbiology, University General Hospital of Alexandroupolis, Alexandroupolis, GRC; 6 Department of Obstetrics and Gynecology, Democritus University of Thrace, Alexandroupolis, GRC

**Keywords:** arabin pessary, cervical cerclage, cervical insufficiency, preterm labor, preventive measures

## Abstract

Objective

The primary objective of this retrospective cohort study was to explore the associations between cervical anatomical features and gestational age at delivery in high-risk asymptomatic singleton pregnancies. A secondary exploratory objective was to evaluate gestational age at delivery according to preventive intervention strategy (Arabin pessary, cervical cerclage, or no intervention).

Study design

This retrospective cohort study included singleton pregnancies managed at a tertiary hospital between December 2019 and December 2024. Eligible pregnancies had documented cervical evaluation (length, diameter, and funneling), intervention status, and gestational age at delivery. The first available cervical measurement was analyzed. Very early preterm birth was defined as delivery before 33+0 weeks to focus on clinically significant prematurity associated with intensive neonatal care.

Methods

Categorical variables (cervical length: ≤2.4 cm vs. >2.4 cm, diameter: ≤10 mm vs. >10 mm, funneling yes/no, and intervention type) were compared using chi-square or Fisher’s exact tests. Gestational age distributions among intervention groups were assessed using kernel density plots and boxplots. Time-to-delivery was evaluated using Kaplan-Meier curves and log-rank testing. Multivariable Cox proportional hazards models included intervention type (reference: cerclage), cervical length, diameter, and funneling. Given the limited number of events, multivariable estimates were interpreted cautiously because of potential overfitting and type II error.

Results

Among 174 singleton pregnancies, 30 (17.2%) delivered before 33 weeks, whereas 144 (82.8%) delivered at ≥33 weeks. Cervical diameter >10 mm was less frequent among pregnancies delivering before 33 weeks (3/30, 10%) compared with those delivering at ≥33 weeks (63/144, 43.8%; p<0.001). Funneling was more frequent in the <33-week group (15/30, 50.0%) than in the ≥33-week group (51/144, 35.4%), although this association did not reach conventional statistical significance in multivariable modeling (p=0.107). Kaplan-Meier analyses suggested longer gestation in the pessary and cerclage groups compared with no intervention, but between-group differences were not statistically significant (log-rank p=0.08). In adjusted Cox models, diameter ≤10 mm was associated with a lower hazard of delivery (HR: 0.398, 95% CI: 0.205-0.433; p<0.001). Other covariates were not statistically significant.

Conclusion

Cervical diameter was associated with gestational age at delivery in this cohort and may represent a candidate marker for risk stratification in high-risk singleton pregnancies. Because of the retrospective design, limited event numbers, and potential indication bias in intervention assignment, these findings should be interpreted as hypothesis-generating rather than definitive evidence of intervention effectiveness or equivalence. Larger prospective and multicenter studies are needed.

## Introduction

Preterm birth (PTB), commonly defined as delivery before 37 weeks of gestation, affects approximately 5-18% of pregnancies worldwide and remains a leading cause of neonatal morbidity and mortality [1]. Among the most important predictors of spontaneous PTB is a shortened cervical length assessed by transvaginal ultrasonography during the second trimester [2]. Even in asymptomatic women with unremarkable first-trimester findings, mid-trimester cervical assessment is considered clinically valuable in the presence of historical or anatomical risk factors for PTB [3]. A cervical length ≤25 mm around 24 weeks of gestation has consistently been associated with increased risk for spontaneous preterm delivery [4].

Additional cervical sonographic findings, including cervical funneling, have also been associated with increased PTB risk, although their independent predictive value beyond cervical length remains controversial [5]. Importantly, not all women with a short cervix ultimately deliver preterm, highlighting the need for more accurate prognostic markers capable of improving risk stratification [6].

Current preventive strategies for PTB include vaginal progesterone, cervical cerclage, and cervical pessary placement. Vaginal progesterone has demonstrated efficacy in reducing PTB risk among women with a shortened cervix, particularly in singleton pregnancies without a previous history of spontaneous PTB [7]. Cervical cerclage is generally recommended in women with prior spontaneous PTB and significant cervical shortening and has been associated with improved outcomes in selected high-risk populations [8]. Evidence also suggests that cerclage may provide greater benefit in cases of extreme cervical shortening, particularly when cervical length is ≤10 mm [9].

The Arabin cervical pessary has emerged as a non-surgical alternative for PTB prevention. Early randomized trials suggested a potential benefit of pessary use in reducing spontaneous PTB [10,11]. However, subsequent studies and meta-analyses have produced conflicting results, with substantial heterogeneity across study populations, cervical length thresholds, and concurrent treatments [12-14]. More recent investigations evaluating combinations of pessary placement and vaginal progesterone have also reported variable effectiveness [15].

Beyond cervical length alone, additional anatomical parameters such as cervical anteroposterior diameter and cervical consistency may contribute to improved PTB prediction. Parra-Saavedra et al. introduced the cervical consistency index (CCI), derived from changes in cervical diameter during ultrasound compression, and demonstrated associations with PTB before 32, 34, and 37 weeks of gestation [16]. These findings support further investigation of cervical structural characteristics beyond simple length measurements.

Despite growing interest in cervical anatomy and preventive interventions, relatively few real-world studies have simultaneously evaluated cervical length, diameter, funneling, and intervention strategies within a time-to-delivery analytical framework in high-risk asymptomatic singleton pregnancies. Therefore, the present retrospective cohort study aimed to explore associations between cervical anatomical features, preventive interventions (cerclage, Arabin pessary, or no intervention), and gestational age at delivery. The primary outcome was delivery before 33+0 weeks of gestation. Given the retrospective design and limited number of preterm events, this study was intended to be exploratory and hypothesis-generating rather than definitive.

## Materials and methods

Study design and setting

We performed a retrospective cohort study at the University Hospital of Alexandroupolis (tertiary care), including singleton pregnancies managed from December 2019 to December 2024. Ethics approval was obtained (#43760/15.10.2021). Data were anonymized prior to analysis. Clinical, ultrasound, intervention, and delivery data were retrospectively extracted from the hospital medical records and entered into an anonymized study database for analysis.

Eligibility criteria

Inclusion and Exclusion Criteria

The inclusion criteria were as follows: (1) singleton pregnancy; (2) documented transvaginal cervical evaluation, including cervical length, cross-sectional diameter, and funneling; (3) known intervention status (Arabin pessary, cerclage, or no intervention); and (4) known gestational age at delivery. The exclusion criteria were as follows: multiple gestation, major fetal anomalies, unavailable cervical parameters or delivery outcomes, and pregnancies that received both interventions.

Missing Data Handling

Because inclusion required complete documentation of cervical parameters, intervention category, and gestational age at delivery, the analytic cohort represents a complete-case subset of the underlying population. This approach may introduce selection bias if excluded cases differ systematically.

Cervical parameters and intervention classification

Cervical length was dichotomized as ≤2.4 cm or >2.4 cm, and cervical diameter (which represents cervical canal width in this study) as ≤10 mm or >10 mm. Funneling was any measurable internal os dilation on transvaginal ultrasound (yes/no). Interventions were categorized as cerclage, Arabin pessary, or no intervention.

Transvaginal cervical ultrasound examinations were performed as part of routine clinical care by experienced obstetrician-gynecologists with expertise in obstetric ultrasonography. Because this was a retrospective study conducted over a five-year period, examinations were performed using the ultrasound equipment routinely available in the department. Cervical length and funneling were assessed using standard transvaginal ultrasound. Cervical diameter was measured on the mid-sagittal transvaginal ultrasound image as the anteroposterior diameter of the cervical canal, obtained perpendicular to the longitudinal axis of the cervix.

The choice of preventive intervention was based on the treating physician's clinical judgment, taking into account obstetric history, cervical ultrasound findings, gestational age, and the overall perceived risk of preterm birth. No standardized institutional protocol for intervention selection was applied during the study period.

Outcome definition

The primary outcome was delivery before 33+0 weeks. This threshold was selected to focus on “very early” preterm birth, which is typically associated with higher neonatal morbidity and resource-intensive care, and to reduce heterogeneity from late preterm deliveries. However, we acknowledge that commonly used clinical thresholds include <37 weeks for preterm birth (PTB) and <34 weeks for “early/severe” PTB; sensitivity analyses using these alternative cutoffs were not performed in this dataset and should be included in future work. Gestational age was determined by first-trimester ultrasound or last menstrual period if an early ultrasound was unavailable.

Statistical analysis

Categorical variables were compared using chi-square or Fisher’s exact tests. Gestational age distributions were visualized with kernel density plots and boxplots. Time-to-delivery was analyzed using Kaplan-Meier survival curves and compared using the log-rank test. Multivariable Cox proportional hazards models included intervention (reference: cerclage), cervical length, diameter, and funneling, reporting HRs with 95% CIs.

Model Robustness and Power Considerations

Given 30 events (<33 weeks) and multiple predictors, the events-per-variable ratio is limited, increasing the risk of overfitting and unstable estimates. Therefore, multivariable results were interpreted cautiously and framed as exploratory. Statistical significance was p<0.05. Analyses were performed using IBM SPSS version 22 (Armonk, NY: IBM Corp.).

Proportional Hazards Assumptions

Formal verification of Cox proportional hazards assumptions (e.g., Schoenfeld residuals or time-by-covariate testing) was not prespecified in the original analysis plan and is not fully documented; this limitation may affect the reliability of time-to-event estimates.

## Results

Study population

A total of 174 singleton pregnancies met the inclusion criteria and were included in the final analysis. Among these, 30 pregnancies (17.2%) resulted in delivery before 33+0 weeks of gestation, whereas 144 pregnancies (82.8%) delivered at or beyond 33 weeks.

Gestational age according to intervention type

**Figure 1:**
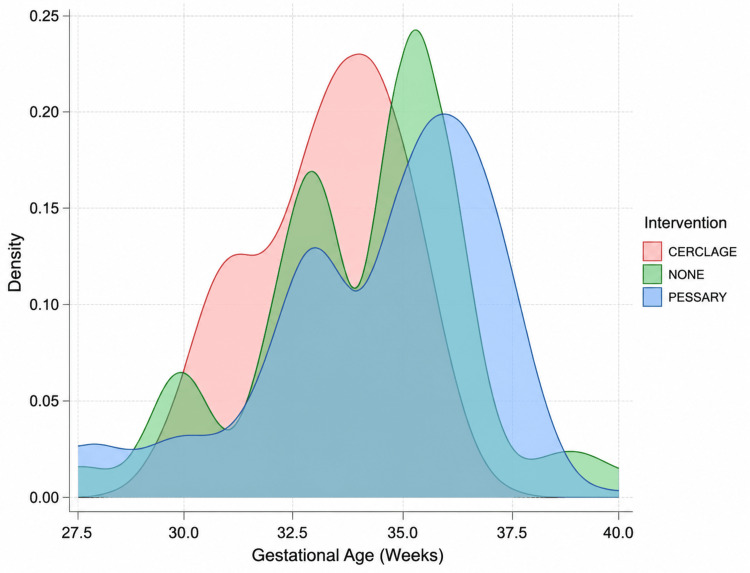
Kernel density plot demonstrating gestational age at delivery according to intervention group. Pregnancies managed with Arabin pessary demonstrated a rightward shift toward longer gestational duration compared with cerclage and no intervention groups.

**Figure 2:**
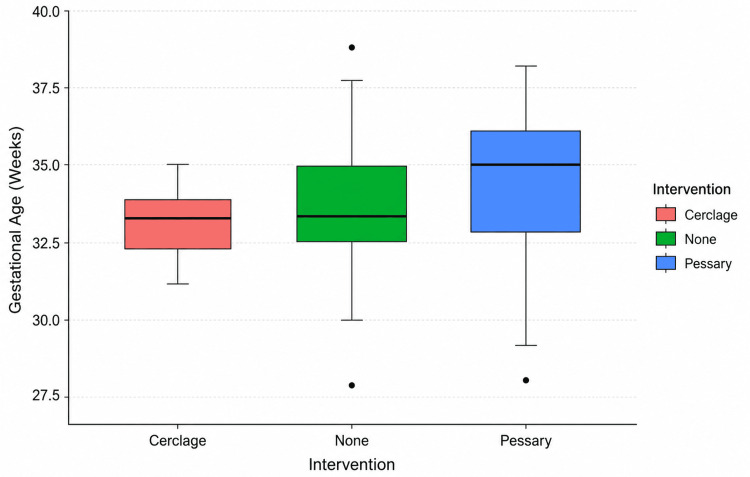
Boxplot comparing gestational age distributions among intervention groups. The pessary group demonstrated the highest median gestational age at delivery and lower variability relative to the cerclage and no intervention groups. Outliers are presented as individual points.

Visual inspection of gestational age distributions using kernel density plots and boxplots demonstrated a trend toward longer gestational duration among pregnancies managed with Arabin pessary placement compared with cerclage or no intervention (Figures 1, 2). The pessary group also appeared to demonstrate lower variability in gestational age at delivery.

However, these observations should be interpreted with caution because intervention allocation was determined clinically rather than randomly, introducing the possibility of confounding by indication and of baseline risk imbalance between groups.

Cervical characteristics and delivery before 33 weeks

Cervical length ≤2.4 cm was observed in 14 of 30 pregnancies (46.7%) delivering before 33 weeks compared with 58 of 144 pregnancies (40.3%) delivering at ≥33 weeks, without a statistically significant association (p=0.655). In contrast, cervical canal diameter (cervical canal width) >10 mm was significantly more common among pregnancies delivering at ≥33 weeks (63/144, 43.8%) than among those delivering before 33 weeks (3/30, 10.0%; p<0.001).

Funneling was identified more frequently in pregnancies delivering before 33 weeks (15/30, 50.0%) compared with pregnancies delivering at ≥33 weeks (51/144, 35.4%). Although this trend suggested increased risk, statistical significance was not maintained in adjusted multivariable analyses. The distribution of cervical characteristics according to delivery outcome is summarized in Table 1.

**Table 1:** Cervical characteristics according to delivery outcome.

Variables	Delivery ≥33 weeks (n=144)	Delivery <33 weeks (n=30)	p-Value
Cervical length ≤2.4 cm, n (%)	58 (40.3%)	14 (46.7%)	0.655
Cervical canal diameter >10 mm, n (%)	63 (43.8%)	3 (10.0%)	<0.001
Funneling present, n (%)	51 (35.4%)	15 (50.0%)	0.107
Pessary use, n (%)	18 (12.5%)	8 (26.7%)	0.066
Cerclage use, n (%)	2 (1.4%)	1 (3.3%)	0.522

Kaplan-Meier time-to-delivery analysis

**Figure 3:**
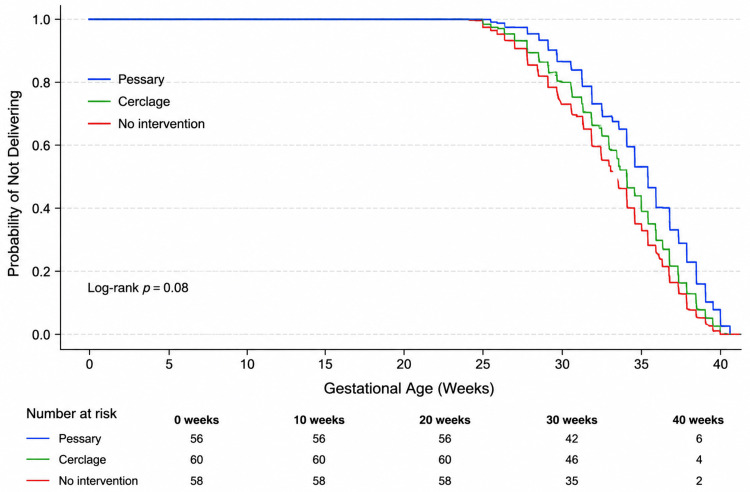
Kaplan-Meier curves illustrating time-to-delivery according to intervention group. Cerclage and pessary groups demonstrated descriptively longer gestational duration compared with no intervention; however, between-group differences were not statistically significant (log-rank p=0.08)

Kaplan-Meier survival analysis demonstrated longer time-to-delivery among pregnancies managed with cerclage or Arabin pessary compared with pregnancies receiving no intervention (Figure 3). Nevertheless, differences between groups did not reach statistical significance using log-rank testing (log-rank p=0.08). Given the retrospective nature of the study, limited event number, and potential confounding by indication, this borderline p-value should not be interpreted as evidence of treatment effectiveness.

Multivariable Cox proportional hazards analysis

Multivariable Cox proportional hazards regression analysis was performed, including intervention type, cervical length, cervical canal diameter, and funneling status (Table 2). Cervical canal diameter ≤10 mm was independently associated with a lower hazard of earlier delivery (HR: 0.398, 95% CI: 0.205-0.433; p<0.001).

**Table 2:** Multivariable Cox proportional hazards model for earlier delivery.

Variables	Hazard ratio (HR)	95% confidence interval	p-Value
No intervention vs. cerclage	0.553	0.284-1.720	0.222
Pessary vs. cerclage	0.344	0.170-1.237	0.155
Cervical length ≤2.4 cm	1.20	0.827-1.618	0.655
Cervical canal diameter ≤10 mm	0.398	0.205-0.433	<0.001
Funneling present	1.357	0.980-1.782	0.107

Neither cervical length ≤2.4 cm nor funneling remained statistically significant predictors after adjustment. Similarly, intervention categories demonstrated non-significant trends with wide confidence intervals, limiting interpretation regarding comparative effectiveness of cerclage and pessary interventions. Because of the relatively small number of preterm events and limited events-per-variable ratio, the multivariable findings should be considered exploratory and hypothesis-generating rather than definitive.

## Discussion

The principal finding of this retrospective cohort study was that cervical diameter demonstrated a significant association with gestational age at delivery before 33 weeks in high-risk asymptomatic singleton pregnancies. Specifically, pregnancies with cervical canal diameter (width) less than 10 mm were more likely to reach later gestational ages compared with those with smaller diameters. Although causality cannot be inferred from this observational design, these findings suggest that cervical diameter may represent a potentially useful anatomical marker for future risk stratification studies.

The role of cervical anatomical assessment in predicting spontaneous preterm birth has been extensively investigated. Cervical length measured by transvaginal ultrasonography remains one of the most established predictors of spontaneous preterm birth [2,4]. However, prior studies have also emphasized that cervical shortening alone may not fully explain the complex pathophysiology underlying preterm delivery [3,6]. In the present study, cervical length ≤2.4 cm was more frequent among pregnancies delivering before 33 weeks, although this association did not remain statistically significant in adjusted analyses. This finding may reflect the limited statistical power of the current cohort and the relatively small number of very early preterm events.

Additional cervical structural parameters may provide complementary predictive value. Parra-Saavedra et al. demonstrated that the cervical consistency index (CCI), derived from cervical anteroposterior diameter changes during ultrasound compression, was associated with spontaneous preterm birth before 32, 34, and 37 weeks of gestation [15]. Although the present study did not directly evaluate CCI, our findings regarding cervical diameter support the broader concept that cervical biomechanical and structural properties may provide additional prognostic information beyond cervical length alone.

Funneling was more commonly observed among pregnancies delivering before 33 weeks; however, this association did not remain independently significant after multivariable adjustment. Previous studies evaluating cervical funneling have reported mixed findings regarding its independent predictive value once cervical length and other anatomical variables are considered [16]. Therefore, the present results are generally consistent with existing literature suggesting that funneling may represent a secondary or supportive sonographic marker rather than an isolated independent predictor.

Preventive interventions for spontaneous preterm birth remain an area of ongoing debate. Vaginal progesterone, cervical cerclage, and Arabin pessary placement are commonly used approaches in selected high-risk populations [6-8]. Cerclage has demonstrated benefit in women with prior spontaneous preterm birth and significant cervical shortening, particularly in cases of severe shortening [7,8]. Similarly, the Arabin pessary has been investigated as a less invasive alternative intervention, although published evidence remains inconsistent.

Early randomized studies suggested the potential benefit of pessary placement in reducing spontaneous preterm birth [9,10]. However, subsequent systematic reviews and meta-analyses have demonstrated substantial heterogeneity across patient populations, cervical length thresholds, and study methodologies [11,12]. More recent investigations, including factorial randomized trials evaluating combinations of cerclage, pessary, and vaginal progesterone, have also produced variable results [14].

In the present cohort, pregnancies managed with cerclage or pessary demonstrated descriptively longer gestational duration compared with pregnancies receiving no intervention. Nevertheless, these differences did not reach statistical significance in Kaplan-Meier or adjusted Cox regression analyses. Importantly, intervention allocation was clinician-driven rather than randomized, introducing a substantial risk of confounding by indication. Women perceived to be at higher baseline risk may have received the intervention preferentially, thereby limiting the ability to infer comparative effectiveness.

Interpretation of the present findings must also consider statistical limitations. The relatively small number of preterm events and limited events-per-variable ratio increase the risk of model overfitting and unstable multivariable estimates. Consequently, non-significant findings should not be interpreted as evidence of the absence of effect or equivalence between interventions. Instead, the results should be considered exploratory and hypothesis-generating.

Despite these limitations, this study contributes clinically relevant observational data by simultaneously evaluating cervical length, cervical diameter, funneling, and intervention strategies within a time-to-delivery analytical framework in high-risk singleton pregnancies. Few real-world studies have incorporated these anatomical and interventional variables concurrently, particularly using survival analysis methodology.

Strengths and limitations

Strengths of this study include detailed ultrasound-based cervical characterization, inclusion of multiple anatomical parameters, and use of time-to-event analyses to evaluate gestational duration. However, several important limitations should be acknowledged. First, the retrospective single-center design limits generalizability and introduces potential selection bias. Second, the intervention assignment was based on clinical judgment rather than on standardized prospective criteria, increasing the risk of confounding by indication. Third, the limited number of preterm events reduced statistical power and increased the risk of overfitting within multivariable models. In addition, the use of delivery before 33 weeks as the primary endpoint limits comparability with studies using conventional thresholds such as <34 or <37 weeks. Finally, formal testing of the proportional hazards assumption was not fully documented and should be incorporated into future prospective analyses.

## Conclusions

Cervical canal diameter more than 10 mm was associated with gestational age at delivery before 33 weeks in this cohort of high-risk singleton pregnancies and may represent a candidate anatomical marker for future risk stratification research. However, because of the retrospective design, limited event number, potential model instability, and confounding by indication, these findings should be interpreted as exploratory rather than definitive evidence regarding the comparative effectiveness of Arabin pessary and cervical cerclage. Larger prospective multicenter studies with standardized intervention criteria, prespecified power calculations, and clinically conventional preterm birth thresholds are required.
